# Secreted Endothelial Cell Factors Immobilized on Collagen Scaffolds Enhance the Recipient Endothelial Cell Environment

**DOI:** 10.1089/biores.2016.0003

**Published:** 2016-03-01

**Authors:** Charlotte Hamilton, Anthony Callanan

**Affiliations:** Institute of Bioengineering, The University of Edinburgh, The King's Buildings, Edinburgh, United Kingdom.

**Keywords:** angiogenesis, biomaterials, gene expression, growth factor, tissue engineering

## Abstract

Strategies to design novel vascular scaffolds are a continuing aim in tissue engineering and often such designs encompass the use of recombinant factors to enhance the performance of the scaffold. The established use of cell secretion utilized in feeder systems and conditioned media offer a source of paracrine factors, which has potential to be used in tissue-engineered (TE) scaffolds. Here we utilize this principle from endothelial cells (ECs), to create a novel TE scaffold by harnessing secreted factors and immobilizing these to collagen scaffolds. This research revealed increased cellular attachment and positive angiogenic gene upregulation responses in recipient ECs grown on these conditioned scaffolds. Also, the conditioning method did not affect the mechanical structural integrity of the scaffolds. These results may advocate the potential use of this system to improve vascular scaffolds' *in vivo* performance. In addition, this process may be a future method utilized to improve other tissue engineering scaffold therapies.

## Introduction

A continuing challenge faced in vascular tissue engineering is how to improve treatments for cardiovascular disease and other such arterial conditions. Current strategies include a number of scaffold materials and/or therapies that mimic the native vessel wall, restore *in situ* endothelialization, promote extracellular matrix (ECM) production, inhibit thrombogenicity, reduce inflammation, and help stimulate neovascularization and angiogenesis.^1,2^ Tissue engineering has utilized a range of biomaterials, including decellularized ECM,^3,4^ synthetic biopolymers,^5^ and biodegradable polymers to create tissue-engineered (TE) vascular grafts.^6–10^

Some of the most common types of scaffold are collagen based^11–13^ and have been shown to promote cell attachment, migration, proliferation, differentiation, and ECM production during remodeling and regeneration.^14^ More recently, they have incorporated growth factors and proteins such as vascular endothelial growth factor (VEGF)^15,16^ and angiopoietin-1 (Ang1).^17,18^ The use of growth factors and proteins has predominately focused on the concentration and release kinetics of these factors, whether they are designed to be retained within the scaffold^19,20^ or released.^21–23^ Mainly, the purpose is to enhance the cell functionality, and contact and interact with the *in vivo* tissue.^24^

In addition, cell secretion can also be a direct or indirect source of paracrine growth factors and proteins. Consequently, this may be one way to partially recapitulate the intrinsic cell environment by using the cell secretion. The principle offered from cell secretion has been utilized directly in the cell culture as cell feeder layer systems to provide paracrine factors to recipient cells.^25^ Cell feeder layers have been widely used to maintain pluripotency of human-induced pluripotent stem cells^26,27^ and human embryonic stem cells.^28,29^ They have also shown promise in tissue regeneration,^30,31^ including secretion from mesenchymal stem cells (MSCs)^32–34^ and endothelial progenitor cells.^35,36^

In this *in vitro* study, we use these principles of the feeder layer cell secretion technique to generate cell conditioned media and incorporate this into a novel TE scaffold. We achieve this by utilizing endothelial cell-secreted factors (ECSFs) and immobilize these to collagen scaffolds and test for improved functionality by the attachment of recipient endothelial cells (ECs).

## Materials and Methods

### Preparation and experimental setup

#### Cell culture

Human umbilical vein endothelial cells (HUVECs) from an infant male Caucasian donor were obtained cryopreserved (500,000 cells) at passage 1 (PromoCell GmbH) and cultured and expanded to passage 5 (P5) in a humidified atmosphere of 5% CO_2_/37°C in T-75 vented flasks (Corning^®^) and grown to 80% confluency. HUVECs were cultured according to a previously used endothelial cell culture protocol,^37–39^ and in brief, MCDB 131 medium (Life Technologies™) was supplemented with 2% fetal bovine serum (FBS; ThermoFisher Scientific); 1% l-glutamine; 1% penicillin/streptomycin (Life Technologies); 1 mg/L hydrocortisone; 50 mg/L of ascorbic acid (Sigma); 2 mg/L fibroblast growth factor; 10 mg/L epidermal growth factor; 2 mg/L insulin-like growth factor; and 1 mg/L VEGF (PeproTech).

##### Basal media

Basal media (BM) consisted of MCDB 131 medium with 2% FBS omitted and all supplements (listed above) added for serum-free cell culture conditions. For experimental conditions, 5 mL of the BM was incubated in a humidified atmosphere of 5% CO_2_/37°C in T-75 vented flasks.

##### Cell conditioned BM

HUVECs at P5 were washed thrice using D-phosphate-buffered saline (PBS)/CaCl_2_ and MgCl_2_ free (Sigma). HUVECs were then cultured in 5 mL of BM for 48 h, incubated in a humidified atmosphere of 5% CO_2_/37°C in T-75 vented flasks, and grown to no more than 70–75% confluency to obtain cell conditioned BM. These media are generally known as cell conditioned media, and for simplicity, further referred to as adjusted BM (ABM). The ABM were filter sterilized using a 0.22-μM filter (Millex^®^ GS Millipore) before use.

#### Collagen scaffolds

Scaffolds discs (10 mm diameter ×2 mm thick) were punched from sheets of commercially available Ultrafoam™ collagen (Davol, Inc.) using a 10 mm disposable biopsy punch (Acuderm, Inc.) on to the surface of a sterile 1.2-mm-thick glass slide (ThermoFisher Scientific). According to the manufacturer's specifications, Ultrafoam is a water-insoluble, partial HCl salt of purified bovine dermal (corium) collagen formed as a sponge with interconnected pores. Collagen scaffolds soaked in D-PBS/CaCl_2_ and MgCl_2_ free (Sigma) served as the control groups for all experiments.

##### Conditioned collagen scaffolds through absorption

**FIG. 1.**
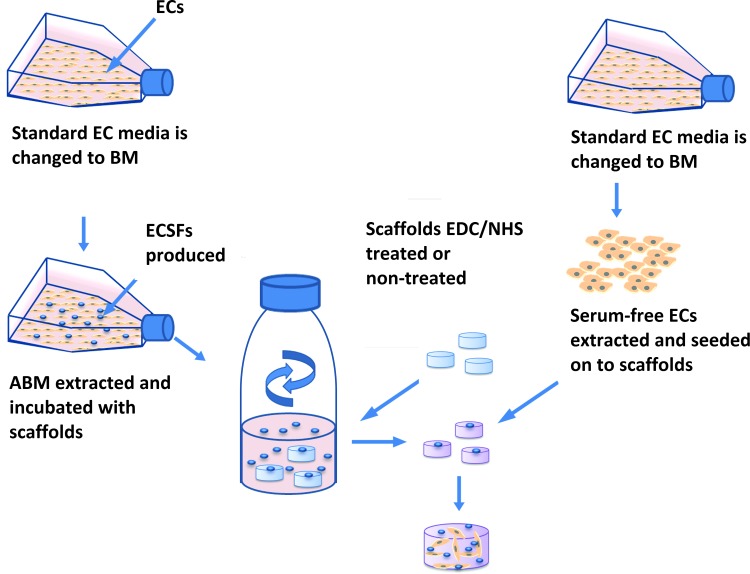
Schematic overview of an example of scaffold preparation using ABM and the generation of autologous ECs to seed on to scaffolds. ECs cultured in standard EC media are switched to BM, this is to generate the ABM from one flask and also to generate autologous serum-free ECs for seeding on to the scaffolds from another flask. The ABM is then incubated with the scaffolds and the autologous ECs are seeded on to the ABM scaffolds. ABM, adjusted basal media; BM, basal media; EC, endothelial cell.

Scaffolds were soaked in PBS, BM, or ABM and incubated 24 h at 37°C and mildly shaken in an orbital shaker (IKA KS 400 i) at 100 rpm in 100-mL Duran flasks during the conditioning process. Refer to Figure 1 for schematic overview of scaffold preparation.

##### Conditioned collagen scaffolds via immobilization

Scaffolds were soaked for 40 min at room temperature with mild agitation in a D-PBS/CaCl_2_ and MgCl_2_ free (Sigma) solution of 1-(3-Dimethylaminopropyl)-3-ethylcarbodiimide—EDC (Sigma) and *N*-hydroxysulfosuccinimide—sulfo-NHS (Sigma; E/N [(1-(3-Dimethylaminopropyl)-3-ethylcarbodiimide and NHS-hydroxysulfosuccinimide)]) at a concentration ratio of 16 mg/24 mg mL^−1^, respectively, and filter sterilized using a 0.22-μM filter. The concentrations used were previously used concentrations for scaffolds.^40^ The scaffolds were then subjected to three successive soaks in fresh D-PBS/CaCl_2_ and MgCl_2_ free (Sigma) for 10 min, each at room temperature with mild agitation to remove any excess E/N. Scaffolds were soaked in PBS, BM, or ABM, incubated for 24 h at 37°C, and mildly shaken at 100 rpm in 100-mL Duran flasks during the conditioning process.

#### Cell seeding

P5 cells were used throughout this study at a seeding density of 5 × 10^5^ cells per scaffold in 100 μL media with *n* = 4 for each condition. For cell seeding, cells were washed thrice using D-PBS/CaCl_2_ and MgCl_2_ free (Sigma) and media changed into BM for 24 h and incubated in a humidified atmosphere of 5% CO_2_/37°C in T-75 vented flasks. Cells were seeded on to the collagen scaffolds in serum-free BM within 12-well nonadherent culture plates (Greiner^®^) and incubated for 1 h at 5% CO_2_/37°C. Unseeded scaffolds in each respective group served as the control. Serum-free BM (1 mL) were then added to cover scaffolds and incubated 24 and 48 h at 5% CO_2_/37°C.

### Experimental quantification

#### Protein quantitation

Media samples were then taken from the six condition groups after 24 h of incubation with scaffolds and before cell seeding. Samples (*n* = 4 scaffolds) were analyzed in quadruplicate using a Protein Quantitation Kit (BioVision^®^) according to the manufacturer's protocol, and the absorbance was measured in clear assay microplates (Greiner) using a 595-nm filter in a Modulus™ II microplate multimode reader.

#### Mechanical testing of collagen scaffolds

Compressional mechanics of collagen scaffolds were accessed to determine the mechanical integrity postmodification due to crosslinking, soaking, and shaking conditions. The compression testing and data interpolation are based on previously used methods for tissue-engineered scaffolds.^41,42^ Scaffolds *n* = 3 were measured in unconfined uniaxial compression testing using an Instron Model 5540 testing machine equipped with a 50-N load cell. The collagen scaffolds were compressed to 60% strain at a strain rate of 0.06 mm/sec. Incremental Young's modulus (i.e., the ratio of stress to strain) was calculated by measuring the slope of the stress–strain plot at incremental strain increases (0–10%, 10–20%, 20–30%, 30–40%, 40–50%, and 50–60%) as previously described.^43^

#### Scanning electron microscopy of collagen scaffolds

Scanning electron microscopy (SEM) characterized the porous architecture of the collagen scaffolds postmodification due to crosslinking and/or soaking, and then shaking conditions. The scaffolds tested, PBS unshaken, PBS shaken, PBE E/N unshaken, and PBS E/N shaken. Scaffolds were snap-frozen and then freeze dried using a FreeZone^®^ 4.5 freeze-drier (Labconco^®^). The samples were then mounted on to metal stubs with double-sided carbon tape. Thin layers of a gold and palladium alloy were applied to each sample with an automated sputter coater (Polaron SputterCoater). The samples were then examined at ×60 low magnification at 5 kV (Hitach S-4700 SEM) as previously shown.^15^

#### CellTiter-blue^®^ cell viability assay

The assay was performed according to the manufacturer's instructions (Promega). For the six condition groups, *n* = 4 scaffolds in duplicate readings to give standard deviation of each group. A range of cell densities were also plated (5 × 10^4^; 10 × 10^4^; 25 × 10^4^; 5 × 10^5^; 7.5 × 10^5^; and 1 × 10^6^ cells/mL^−1^) and counted to give a proportional ratio of cell number: fluorescence emitted within this standard curve. Samples were analyzed in a Modulus II microplate multimode reader using a filter of 525 nm Ex/580–640 nm Em.

#### Live/dead^®^ viability/cytotoxicity assay

This assay was performed according to the manufacturer's protocol (Molecular Probes™ Life Technologies) for fluorescence microscopy on the seeded scaffolds. The working concentration of the calcein AM and EthD-1 dyes was diluted to 0.2 and 0.4 μM, respectively, from the suggested working concentrations of 2 and 4 μM, respectively. Scaffolds were washed thrice to remove excess dye in D-PBS/CaCl_2_ and MgCl_2_ free (Sigma) and placed on a well slide with 25 mm cover-slip (Scientific Laboratory Solutions). Microscopy was performed using a Zeiss Axio Imager fluorescent microscope using a 40× objective.

#### DNA quantitation

Cell-seeded scaffolds after the 24- and 48-h growth periods were snap-frozen and stored at −20°C. Scaffolds were then freeze-dried overnight using a FreeZone 4.5 freeze-drier (Labconco) to remove any residual water content before DNA extraction. The scaffolds were then digested in a solution of D-PBS/CaCl_2_ and MgCl_2_ free (Sigma), containing 2.5 U/mL papain extract (Sigma), 5 mM cysteine-HCl (Sigma), and 5 mM EDTA (Sigma), and samples were incubated overnight at 60°C. Cell extracts (*n* = 4) of 5 × 10^5^ cells frozen at −20°C when scaffolds were seeded, served as the control. Samples (*n* = 4 scaffolds) were mixed thoroughly before assay. A Quant-IT™ PicoGreen^®^ dsDNA Assay Kit (Life Technologies) was used and performed according to the manufacturer's protocol based on 200 μL volume for microplate reader analysis. Samples were analyzed in a Modulus II microplate multimode reader using a filter of 490 nm Ex/510–570 nm Em.

#### RNA isolation

Cell-seeded scaffolds after the 24- and 48-h growth periods were snap-frozen in 350 μL TRIzol^®^ (Sigma) and stored at −80°C until preparation. On thawing, the scaffolds were homogenized using a TissueRuptor™ device (Qiagen) and centrifuged at 12,000 rpm to obtain an aqueous layer and this was subjected to a chloroform extraction and 70% ethanol precipitation. The RNA was then prepared using an RNeasy^®^ kit (Qiagen) according to the manufacturer's protocol. The RNA (100 ng/μL) was used to prepare cDNA using ImProm-II™ Reverse Transcription System (Promega) according to the manufacturer's instructions.

#### Quantitative reverse transcription-polymerase chain reaction

The quantitative reverse transcription-polymerase chain reaction (qRT-PCR) was performed in triplicate using three independent cDNA samples with additional respective RT- samples to investigate gene expression after seeding on scaffolds. SensiFAST™ SYBR^®^ Hi-ROX (Bioline) was used in the reaction and the reaction was performed using a LightCycler^®^ 480 Instrument II (Roche Life Science) for standard program of 45 cycles. Relative quantification of the RT-PCR results was carried out using the 2^^−ΔΔct^ method.^44,37^ Forward and reverse primer sequences (Sigma) were as follows: Glyceraldehyde 3-phosphatedehydrogenase (*GAPDH*): forward primer ′5-GTCTCCTCTGACTTCAACAG-3′, reverse primer, ′5-GTTGTCATACCAGGAAATGAG-3′; vascular endothelial growth factor A (*VEGFA*): forward primer ′5-AGACCAAAGAAAGATAGAGCAAGACAAG-3′, reverse primer ′5-GGCAGCGTGGTTTCTGTATCG-3′; matrix metalloproteinase 1 (*MMP1*): forward primer ′5-AGCTAGCTCAGGATGACATTGATG-3′, reverse primer 5′-GCCGATGGGCTGGACAG-3′; von Willebrand factor (*vWF*): forward primer 5′-GCAGTGGAGAACAGTGGTG-3′, reverse primer 5′-GTGGCAGCGGGCAAAC-3′; *Ang1*: forward primer 5′-ATTCTGAATGGTGGGGAGCA-3′, reverse primer 5′- TGTGCTGGGATGGGAAAGAT-3′; platelet/endothelial cell adhesion molecule (*PECAM/CD31*): forward primer 5′- ATTGCAGTGGTTATCATCGGAGTG-3′, reverse primer 5′-CTCGTTGTTGGAGTTCAGAAGTGG-3′; and tissue inhibitor of matrix metalloproteinase-2 (*TIMP2*): forward primer 5′-AATGCAGATGTAGTGATCAGG-3′, reverse primer 5′-TCTATATCCTTCTCAGGCCC-3′.

### Statistical analysis

Data are presented as average ± standard error mean. Statistical significance was determined by performing one-way ANOVA with *n* = 4 for protein quantitation assay, CellTiter-blue cell viability assay, and the DNA quantitation assay. For qRT-PCR, *n* = 3 and for compression testing, *n* = 3. All data presented with significance accepted *p* < 0.05.

## Results

### Evaluation of scaffold properties

#### Retainment of ECSFs

**FIG. 2.**
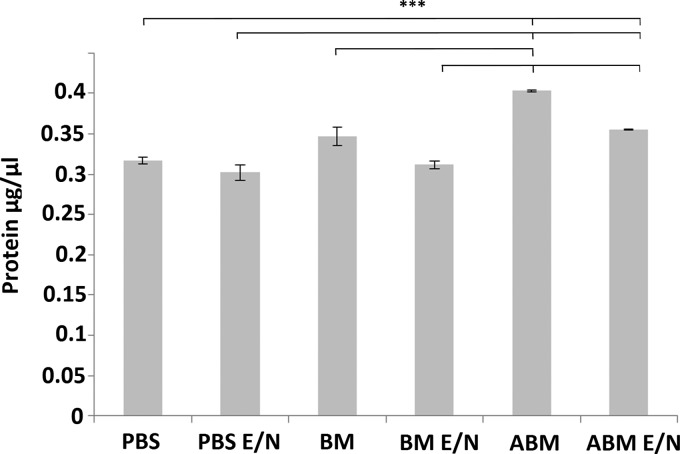
Protein quantitation of media samples 24 h postscaffold incubation and before cell seeding. Statistical significance between groups denoted by ***p < 0.01.

The level of protein released into or extracted from different conditioning fluids was investigated. The protein concentration in the conditioning fluids was measured after agitation with the scaffolds to show the influence in processing methods. The protein released into the PBS group had an average concentration of 0.32 μg/μL (as the collagen scaffold itself is a source of protein), while in the BM group this was higher at 0.35 μg/μL, due to the additional components present within the media. In addition, the ABM group had the largest concentration at 0.4 μg/μL, with the presence of ECSFs in the media. In the functionalized groups, which showed significant difference between PBS E/N (0.3 μg/μL) and BM E/N (0.31 μg/μL), these displayed 25% and 22.5% less protein compared to the ABM group, respectively. The surface functionalization step demonstrated that the ECSFs were retained in the scaffolds, as shown by the reduction of free protein constituents found in the media (conditioning fluid) observed for these groups, with the ABM E/N group (0.35 μg/μL) having 12.5% less in the conditioning fluid compared to the (nonfunctionalized) ABM scaffolds, with a similar trend being shown in the other respective groups (Fig. 2).

#### Scaffold integrity

**FIG. 3.**
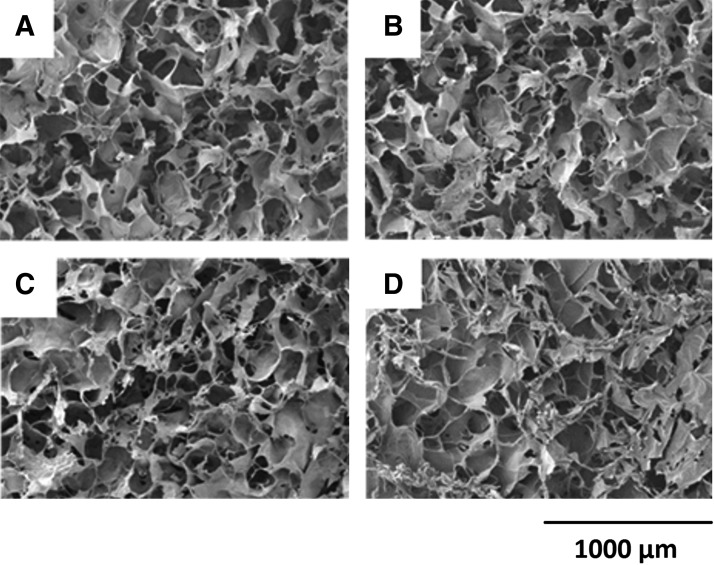
Scanning electron microscopy images of collagen scaffolds postmodification. **(A)** PBS unshaken; **(B)** PBS shaken; **(C)** PBS E/N unshaken; **(D)** PBS E/N shaken. E/N, (1-(3-Dimethylaminopropyl)-3-ethylcarbodiimide and NHS-hydroxysulfosuccinimide); PBS, phosphate-buffered saline.

In these experiments, we assessed the effect of postmodification of the scaffolds, due to soaking and/or E/N surface functionalization within agitation conditions and to determine if the mechanical integrity was modified. The compression properties of the collagen scaffolds were tested, as was the corresponding surface topography analyzed by SEM. Large differences in Young's modulus were seen at low strain intervals (10–20%) with a maximum of 50% difference observed between PBS shaken (0.4 kPa) and unshaken (0.20 kPa). At a high strain interval of 50–60%, the compression difference between PBS shaken (3.08 kPa) and PBS unshaken (2.78 kPa) was reduced to 9.7% difference. No statistical significant differences were shown between the groups throughout the intervals of the Young's modulus (Table 1.). The surface topography between the scaffold groups showed no vast difference in the macroporous or microporous structure of the collagen (Fig. 3.) with pore sizes varying between 50–200 μm approximately across the surface.

**Table 1.** **Scaffold Compression Displaying Young's Modulus (kPa) Among Four Scaffold Conditions**

	% Compression											
	0–10		10–20		20–30		30–40		40–50		50–60	
Young's modulus (kPa)	Mean	SD	Mean	SD	Mean	SD	Mean	SD	Mean	SD	Mean	SD
PBS unshaken	0.20	0.20	0.62	0.07	1.01	0.04	1.13	0.12	1.62	0.27	2.78	0.60
PBS shaken	0.40	0.18	0.71	0.09	1.05	0.11	1.29	0.16	1.79	0.32	3.08	0.79
PBS E/N unshaken	0.32	0.10	0.81	0.30	1.18	0.01	1.26	0.19	1.58	0.23	2.28	0.58
PBS E/N shaken	0.22	0.22	0.57	0.36	1.02	0.12	1.20	0.14	1.59	0.32	2.29	0.72

E/N, (1-(3-Dimethylaminopropyl)-3-ethylcarbodiimide and NHS-hydroxysulfosuccinimide); PBS, phosphate-buffered saline; SD, standard deviation.

### Assessment of scaffold functionality

#### EC attachment and viability

**FIG. 4.**
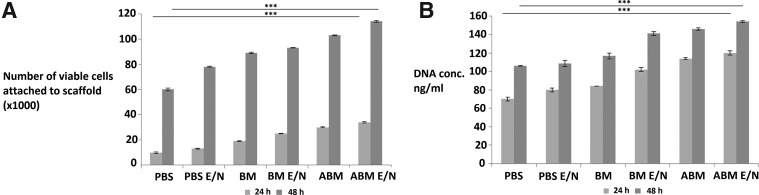
The number of viable cells attached to scaffolds **(A)**; DNA quantification of scaffolds **(B)** at 24 and 48 h postcell seeding. Statistical significance between groups denoted by ***p < 0.01.

**FIG. 5.**
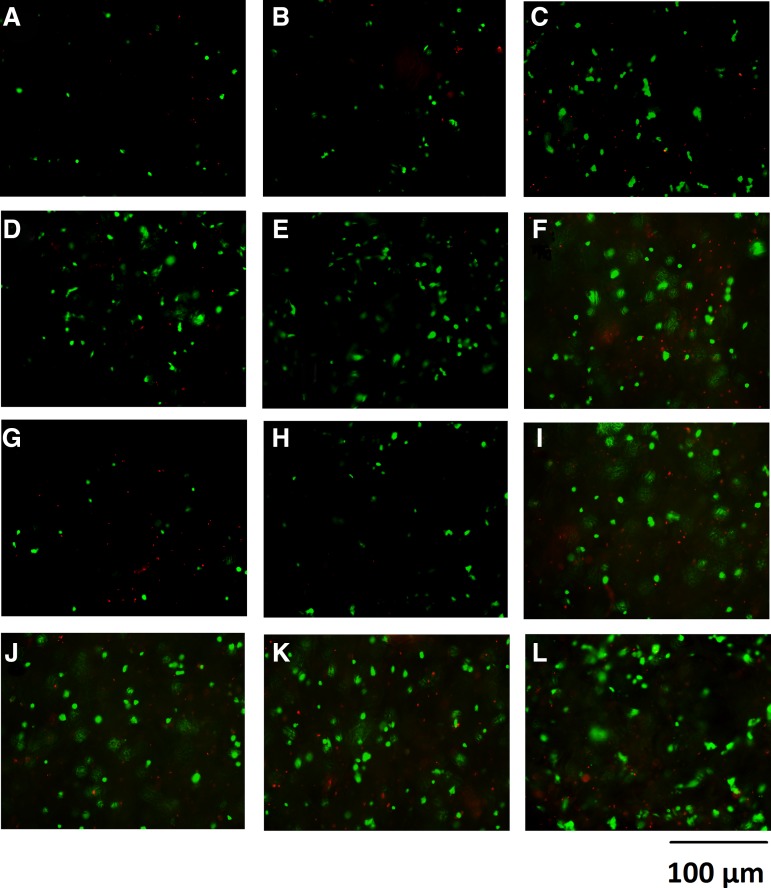
Live/Dead^®^ Viability/Cytotoxicity assay of ECs bound to collagen scaffolds at 24 h postcell seeding: **(A)** PBS; **(B)** PBS E/N; **(C)** BM; **(D)** BM E/N; **(E)** ABM; **(F)** ABM E/N. Then, at 48 h postcell seeding: **(G)** PBS; **(H)** PBS E/N; **(I)** BM; **(J)** BM E/N; **(K)** ABM; **(L)** ABM E/N. Green cell staining represents calcein AM dye corresponding to viable cells, red cell staining represents Ethd-1 dye and corresponds to apoptotic or dead cells. Magnification used ×40 objective lens and scale bar represents 100 μm.

The ability of cells to attach to the scaffolds was assessed and also their viability once attached to the scaffolds (Fig. 4A). The number of attached viable cells (NAVC) after 24 h displayed a significant progressive increase across the six scaffold conditions with ABM scaffolds (30 × 10^3^ cells attached) compared to 33% and 10% less attached cells in BM and PBS scaffolds, respectively. The E/N treated scaffolds showed a greater NAVC than untreated scaffolds across all groups. The NAVC were most profound with ABM E/N scaffolds (35 × 10^3^ cells attached) compared to 71% less attached cells in PBS scaffolds (10 × 10^3^ cells attached). However, at 48 h there was an increase in the NAVC, which was significantly greater in all these scaffold groups, with the ABM E/N scaffolds (110 × 10^3^ cells attached) compared to 45% less cells attached in PBS E/N scaffolds. Comparing the difference between the 24- and 48-h values of NAVC within the six conditions, the level increased exponentially. The amount of DNA retained on the scaffolds from cell attachment showed the same trend across the scaffold groups at 24 h. This was then further increased within these scaffold groups at 48 h, with ABM E/N showing the greatest concentration of DNA retained on the scaffold at 1500 ng/mL (Fig. 4B). However, the difference in DNA concentration between the groups at 24 and 48 h is not exponential unlike the NAVC (described above). Visual microscopy using Live/Dead Viability/Cytotoxicity assay showed NAVC on the scaffolds, with the greatest amount on the ABM E/N scaffolds (Fig. 5).

#### Gene expression of ECs on scaffolds

**FIG. 6.**
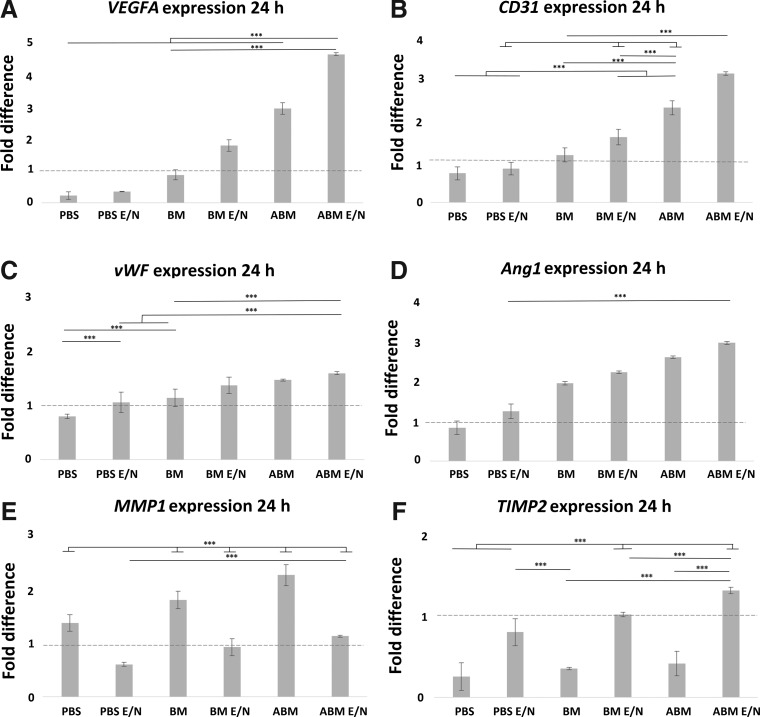
The qRT-PCR of mRNA expression of genes: **(A)** *VEGFA;* **(B)** *CD31;* **(C)** *vWF;* **(D)** *Ang1;* **(E)** *MMP1;* **(F)** *TIMP2* at 24 h postcell seeding on collagen scaffolds. The mRNA expression of these genes (y-axis) was normalized to *GAPDH* and relative to an EC cDNA-positive control expression (dotted line). Error bars represent ±1 SD, *n* = 3 of delta cT value. *Ang1*, angiopoietin-1; *MMP1*, matrix metalloproteinase 1; qRT-PCR, quantitative reverse transcription-polymerase chain reaction; SD, standard deviation; *TIMP2*, tissue inhibitor of matrix metalloproteinase-2; *VEGFA*, vascular endothelial growth factor A; *vWF*, von Willebrand factor.

**FIG. 7.**
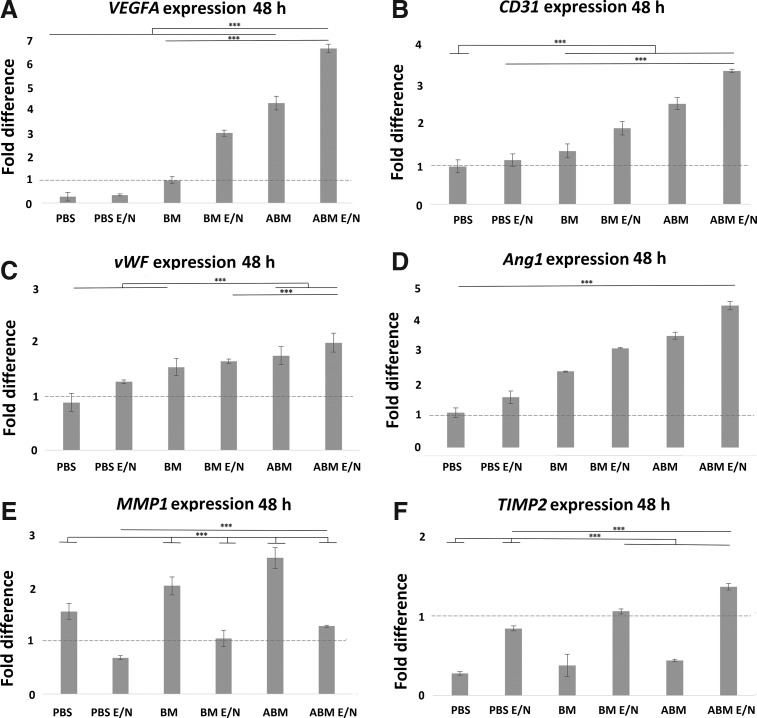
The qRT-PCR of mRNA expression of genes: **(A)** *VEGFA;* **(B)** *CD31;* **(C)** *vWF;* **(D)** *Ang1;* **(E)** *MMP1;* **(F)** *TIMP2* at 48 h postcell seeding on collagen scaffolds. The mRNA expression of these genes (y-axis) was normalized to *GAPDH* and relative to an EC cDNA-positive control expression (dotted line). Error bars represent ± 1 SD, *n* = 3 of delta cT value.

The analysis of gene expression accessed the functionality of the recipient ECs (seeded) and determined if the scaffold preparation method effected the EC response in terms of expression of key angiogenic and regulatory genes (Figs. 6 and 7). The results indicate a progressive increase in notably *VEGFA* and *Ang1*, across all scaffold groups, with ABM scaffolds showing the greatest increase of gene expression, further enhanced by E/N surface functionalization. This ABM E/N group showed the greatest level of expression compared to the lowest level with PBS scaffolds and this was significantly sixfold and threefold higher in *VEGFA* and *Ang1*, respectively, at 24 h. These levels increased 6.5-fold higher and 3.5-fold higher at 48 h. The key functional gene *CD31* was also increased in all groups, with the greatest level in the ABM E/N scaffolds. For the same comparison with PBS scaffolds, the levels were threefold higher at 24 h and significantly 2.5-fold higher at 48 h. The *vWF* expression showed marginal differences when compared across groups, with the largest increase (twofold higher) in PBS scaffolds between the 24- and 48-h time point. *MMP1* was significantly twofold lower in the E/N functionalized scaffolds at 24 and 48 h when compared to untreated scaffolds. Conversely, the expression of *TIMP2*, showed a significant increase (twofold) in the E/N scaffolds at 24–48 h.

## Discussion

The majority of recent studies have used the incorporation of recombinant factors to enhance the performance of TE scaffolds for specific treatments. This can often be complex with varying success, especially when multifactors are used.^45^ An alternative approach to produce the growth factors or proteins is by the use of cell secreted factors. This has been previously achieved by one of the two methods: by the use of cell feeder layers or by obtaining conditioned media. One successful strategy used the MSC-derived conditioned medium that promoted proliferation of cardiac progenitor cells, inhibited apoptosis induced by hypoxia and serum starvation, and, furthermore, upregulated expression of a cardiomyocyte-related gene.^46^ This strategy of paracrine cell secretion has been utilized in many regenerative medical applications, namely with the use of stem cells, through which the paracrine secretion from these cells elicits a response in recruitment of host cells to the tissue environment.^47,48^

In this study, we generated a conditioned media in a bovine-free serum containing the endogenous ECSFs and attached this to the scaffold using a number of techniques. This process demonstrated an increase in the level of protein present initially by conditioning scaffolds using BM but more so by using a cell conditioned media (ABM) scaffold. By using a surface crosslinking reaction, we were able to retain and further enhance the conditioned scaffolds and show a greater significant effect in viable cell attachment when these scaffolds have the presence of ECSFs. Nevertheless, the crosslinking reaction served to increase the retention of bound factors upon agitation of BM and ABM scaffold conditions and even enhanced the performance of PBS scaffolds. The further benefits of also using a crosslinking approach such as E/N, proved not only to unaffect the collagen structural and mechanical integrity but to also slightly enhance the stability of any collagen degradation at 48 h.^49^ E/N crosslinking has been widely used in the immobilization of recombinant growth factors to collagen scaffolds,^15–17^ but here we were also able to demonstrate a stable sustained effect when immobilizing media.

The key finding was that we determined an enhancement effect from this scaffold modification method. This was initially observed using BM and became more profound when ABM scaffolds were used and then further increased by functionalizing with E/N, displaying significant differences between the groups tested. To represent the phenotype from the attached recipient cells, key gene expression was evaluated. There was also an unaltered endothelial phenotypic response from the attached autologous cells in serum-free conditions; however, there was an enhanced effect in angiogenic genes. In addition, we were able to show that the collagen integrity was stable between 24 and 48 h, as *MMP1* representing collagen degradation was reduced when scaffolds were E/N treated. Likewise, the inverse expression of *TIMP2* representing collagen integrity was increased when scaffolds were E/N treated.

While the preliminary findings of this study are promising, there are important limitations and other parameters that exist should be considered. An important limitation is in the scaffold type used, which does not have potential as a vascular substitute, however, there is potential to use this novel conditioning process on other scaffolds types. In addition, there are limitations in the process used to produce the conditioned media, which could be modified to adjust the secreted factors produced. A number of mechanisms could be used to achieve this, such as modification to the serum-free culture^50^ or by exploiting hypoxic conditions to over produce secreted factors.^51^ Furthermore, the long-term activity could be investigated to access the potential for an off-the-shelf scaffold approach using this processing technique. Nevertheless, these studies have shown the potential of a cell secretion method for scaffold applications and also provide this method within a serum-free environment.

## Conclusions

In this study, we have demonstrated a scaffold model utilizing a novel cell secreted method for specifically ECs. Taken together, our results and the core principle of this method highlight the potential that could be extended to other cell types, tissue environments, and suitable scaffold materials in tissue engineering and regenerative medicine applications. This strengthens the case for its potential as a translatable clinical process for improvement in scaffold performance.

## Abbreviations Used

ABM: adjusted basal media
Ang1: angiopoietin-1
BM: basal media
CPC: cardiac progenitor cells
E/N: (1-(3-Dimethylaminopropyl)-3-ethylcarbodiimide and NHS-hydroxysulfosuccinimide)
ECM: extracellular matrix
ECs: endothelial cells
ECSFs: endothelial cell-secreted factors
FBS: fetal bovine serum
HUVECs: human umbilical vein endothelial cells
MMP1: matrix metalloproteinase 1
MSCs: mesenchymal stem cells
NAVC: number of attached viable cells
P5: passage 5
PBS: phosphate-buffered saline
SD: standard deviation
TIMP2: tissue inhibitor of matrix metalloproteinase-2
VEGF: vascular endothelial growth factor
vWF: von Willebrand factor
